# Long-Term Exposure to Temozolomide Affects Locomotor Activity and Cartilage Structure of Elderly Experimental Rats

**DOI:** 10.3390/biomedicines8120541

**Published:** 2020-11-26

**Authors:** Anastasia V. Suhovskih, Olga P. Molodykh, Victor S. Ushakov, Maxim O. Politko, Dmitry K. Sokolov, Elena V. Koldysheva, Elvira V. Grigorieva

**Affiliations:** 1 Department of Molecular Biology and Biophysics, Federal Research Center of Fundamental and Translational Medicine, 2/12, Timakova str., 630117 Novosibirsk, Russia; v.ushakov.nsu@gmail.com (V.S.U.); politko.nsu@gmail.com (M.O.P.); dmit_s95@mail.ru (D.K.S.); elvira.grigorieva@niimbb.ru (E.V.G.); 2 Department of Natural Sciences, V. Zelman Institute for Medicine and Psychology, Novosibirsk State University, 1, Pirogova str., 630090 Novosibirsk, Russia; 3 Department of Molecular Pathology and Pathomorphology, Federal Research Center of Fundamental and Translational Medicine, 2, Timakova str., 630117 Novosibirsk, Russia; sib_plus@mail.ru (O.P.M.); 130066@mail.ru (E.V.K.)

**Keywords:** temozolomide, dexamethasone, locomotor activity, cartilage morphology, glycosaminoglycan content

## Abstract

Chemotherapy with temozolomide (TMZ) is an essential part of anticancer therapy of various malignant tumours; however, its long-term effects on patients’ health and life quality need to be further investigated. Here, we studied the effects of TMZ and/or companion drug dexamethasone (DXM) on the locomotor activity and cartilage structure of elderly Wistar rats (*n* = 40). Long-term TMZ treatment selectively inhibited the horizontal, but not vertical locomotor activity of the rats (6.7-fold, *p* < 0.01) and resulted in delamination of the superficial epiphyseal cartilage of the femoral epiphysis of knee joints, a 2-fold decrease in mean thickness of epiphyseal cartilage (*p* < 0.001), and changes in the proliferative and maturation cartilage zones ratio. The simultaneous use of DXM attenuated TMZ-induced changes in cartilage thickness and integrity and compensated the decrease in horizontal locomotor activity of experimental animals. Nevertheless, combined TMZ/DXM treatment still significantly affected the structure of proximal tibial, but not distal femoral epiphysis of knee joints of the rats. These changes were accompanied by the increased content of total glycosaminoglycans (GAGs) and their partial re-localisation from chondrocytes into tissue matrix, as well as the decrease in sulfated GAGs content in both compartments. Taken together, the results demonstrate that long-term treatment with TMZ results in a significant decrease in locomotor activity of elderly Wistar rats and the reorganisation of their knee joint cartilage structure, while DXM treatment attenuates those effects. So, use of DXM or chondroprotective drugs might be beneficial to maintain quality of life for TMZ-treated cancer patients.

## 1. Introduction

Temozolomide (TMZ) is a conventional chemotherapy drug for treating various malignant tumours such as melanoma [1], metastatic adrenocortical carcinoma [2], prolactin-secreting adenoma, and carcinoma [3]. Thanks to its ability to cross the blood–brain barrier, TMZ is widely used for brain tumours [4,5], other cancers of the central nervous system [6], and brain metastases from other malignancies [7,8]. However, the use of TMZ leads to many adverse side effects such as myelosuppression, non-haematologic toxicity and infections, and resistance development to the chemotherapeutic drug [9]. The most common haematological side effects are anaemia, leucopenia, and thrombocytopenia [10,11], while non-haematological manifestations include fatigue, nausea, and vomiting [12,13].

Data on long-term TMZ effects on health-related quality of life and cognitive function are quite controversial and demonstrate both no negative side effects during the chemotherapy [14,15] and significant depression starting at the third month after adjuvant TMZ therapy [16]. It was shown that mice treated with a regimen of TMZ similar to humans demonstrate anxiety- and depression-like behaviour [17] and behavioural and biochemical changes that have relevance to the development of depression [18]. Another important parameter of a high life quality after TMZ treatment is physical activity that might be compromised by TMZ chemotherapy. It has been shown that short-term TMZ treatment does not affect locomotion of adult mice [18,19], although in combination with matrix metalloproteinase inhibitor marimastat, it led to joint and tendon pain in 47% of enrolled glioblastoma patients in a clinical trial [20]. However, the long-term effects of TMZ administration on locomotor activity, especially in the elderly, remain unknown. Nevertheless, it cannot be ruled out that long-term use of TMZ affects motor activity, thereby reducing the life quality.

Because of the fact that, during anticancer chemotherapy, TMZ is used usually in combination with glucocorticoid drug dexamethasone (DXM) to prevent neurological symptoms, treatment-related vasogenic oedema, and increased intracranial pressure [21,22,23], it is of importance to take into account the contribution of this component as well. In mono-regimen, long-term treatment with steroids results in adrenal insufficiency, increased infection risk, hyperglycaemia, high blood pressure, osteoporosis, development of diabetes mellitus, peripheral oedema, and psychiatric disorders [21,24]. DXM treatment also affects cognitive function, as the children exposed to DXM during the first trimester had an impaired verbal working memory [25]; however, the observed deficits in executive functioning during childhood may improve with time [26]. DXM-treated mice exhibited a variety of depression-like behaviour and increased anxiety-like behaviour in a dose-dependent manner [27,28]. It was shown that DXM also decreases the locomotor activity of neonatal rats [29] and adult animals [30,31]. Long-term effects of DXM treatment have not been investigated yet, as well as the effects of combined therapy with TMZ and DXM.

This work aims to study the effects of long-term TMZ and/or DXM administration on the motor activity of elderly rats and morphological changes in the knee joints of the animals in an experimental model in vivo.

## 2. Experimental Section

### 2.1. Animals

Nine-month-old male Wistar rats weighing 400–500 g at the beginning of the experiment were used (total of 40 animals). Animals were obtained at the Institute of Cytology and Genetics (ICG SB RAS, Novosibirsk, Russia). Animals were housed in polycarbonate cages (36 × 50 × 28 cm) with free access to food and water, temperature of 25 ± 1 °C, humidity of 50–60%, weighed once/day, and 12 h/12 h light/dark cycle. Animals were sacrificed by decapitation using guillotine according to the AVMA Guidelines for the Euthanasia of Animals (American Veterinary Medical Association, 2013). All efforts were made to minimise animal suffering and to reduce the number of animals used. All procedures were conducted in accordance with European Communities Council Directive 2010/63/EU and in compliance FRC FTM Ethical committee.

### 2.2. TMZ and DXM Administration

Rats were randomly divided into four groups (control, TMZ, DXM, and TMZ/DXM; 10 animals/group). A TMZ-based drug (Temozolomide-Teva) was administered peroral 150 mg/m^2^ per day, and the synthetic glucocorticoid agonist DXM (KRKA) was administered subcutaneously 2.5 mg/kg. Animals received five cycles of TMZ and/or DXM for 5 consecutive days, with breaks of 16 days between cycles. The control group received saline injections/peroral administration of the same volume as the experimental groups. Animals were sacrificed by decapitation and knee-joint samples from each animal were collected.

### 2.3. Open Field Test

The experimental animals were tested before the start of the experiment at the age of 9 months and after the treatments at the age of 13 months. The rats were placed on the open-field arena (100 × 100 × 40 cm) and allowed to freely explore the arena for 5 min. Rat behaviour: grooming, rearing and crossings number, and boli were monitored using a Canon PowerShot G10 camera and manual counting for 5 min.

### 2.4. Histological Analysis

To study the morphology of the articular cartilage of rats, fragments of knee joints were fixed in 10% neutral formalin; decalcified in a mixture of 90% formic acid, concentrated hydrochloric acid, and distilled water (in the proportion of 10:8:82) for 4–6 days; washed in 96% ethanol and distilled water; and embedded in paraffin. Serial 3–4 µm sections were stained with haematoxylin and eosin in combination with Pearls reaction. The tissues were analysed using light microscope LeicaDM 4000B (Leica, Wetzlar, Germany) with the LeicaDFC 320 camera (Leica, Germany) and a measurement software program LeicaQWin V3 (Leica, Germany).

### 2.5. Staining for Total and Sulfated Glycosaminoglycans

To determine the content of total and sulfated glycosaminoglycans in the tissue samples, Alcian blue pH 2.5–1.0 Kit (Biovitrum, St. Petersburg, Russia) was used. Briefly, Alcian blue staining of the tissue sections at different pH allows discriminating acid mucopolysaccharides (pH 2.5) and polysulfate mucopolysaccharides (pH 1). Sections were deparaffinised in the series of xylene and rehydrated in ethanols, stained in the Alcian blue solution for 30 (pH 2.5) and 10 (pH 1) min, incubated with sodium tetraborate for 30 min, respectively. The slides were rinsed in distilled water, counterstained in haematoxylin, dehydrated, and mounted.

### 2.6. Statistical Analysis

Statistical analysis of the obtained data was performed with analysis of variance (ANOVA) with Fisher’s post-hoc test using ORIGIN 8.5 software (OriginLab Corporation, Northampton, MA, USA); a value of *p* < 0.05 was considered to indicate a statistically significant difference. Data are expressed as the mean ± SD.

## 3. Results

### 3.1. Long-Term Administration of TMZ and DXM Affects Locomotor Activity of Wistar Rats

**Figure 1:**
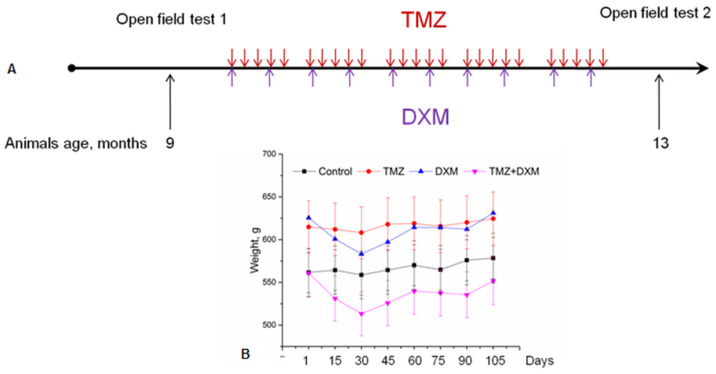
Experimental design of the study. (**A**) Scheme of TMZ and DXM administration to the Wistar rats. (**B**) Weight of experimental animals. All data are expressed as mean ± SD (ORIGIN 8.5 software). TMZ—temozolomide, DXM—dexamethasone.

To study the effects of long-term treatments with TMZ and/or DXM on the behaviour of elderly Wistar rats (9 months old), the animals were tested in the open field arena before and after the treatments. Animals received dosages of drugs, the most appropriate to those for humans. To mimic the cyclic treatment administered in the clinic, the animals were treated with five treatment cycles (5 consecutive days every week with breaks of 16 days between cycles), for 15 weeks in total (Figure 1A).

**Figure 2:**
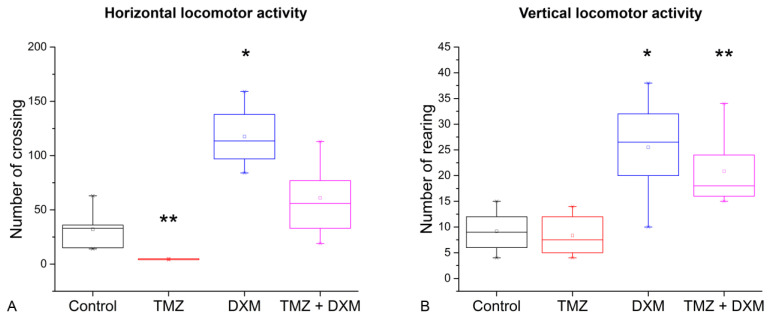
Locomotor activity of rats in the open field test after TMZ and/or DXM treatment. (**A**) Horizontal locomotor activity (number of square crossing). (**B**) Vertical locomotor activity (number of rearing). * *p* < 0.05, ** *p* < 0.01 compared with control. All data are expressed as mean ± SD (ORIGIN 8.5 software). TMZ—temozolomide, DXM—dexamethasone.

During the experiment, the weight of the animals was controlled. The observed weight fluctuations were not pronounced and indicated the absence of excessive toxicity of the drugs used (Figure 1B). The long-term treatments with TMZ and/or DXM did not significantly affect the grooming activity or the number of faecal boli in the open-field test, indicating that these drugs do not affect anxiety in the elderly experimental rats in this test. However, both TMZ and DXM specifically affected the locomotor activity of the rats (Figure 2).

TMZ-treated rats demonstrated a significant decrease in their horizontal locomotor activity (6.7-fold, *p* < 0.01), but there were no significant differences in vertical locomotor activity compared with the control rats. At the same time, DXM treatment significantly stimulated both horizontal (3.7-fold) and vertical (2.8-fold) locomotor activities of the rats (*p* < 0.05), and compensated the TMZ-induced decrease in horizontal locomotor activity (Figure 2A). In general, the results support the hypothesis of a possible influence of long-term TMZ treatment on locomotor activity of elderly animals and demonstrate the protective effect of DXM to these side effects of TMZ.

### 3.2. TMZ Affects Epiphyseal Cartilage Morphology in Rat Knee Joints

**Figure 3:**
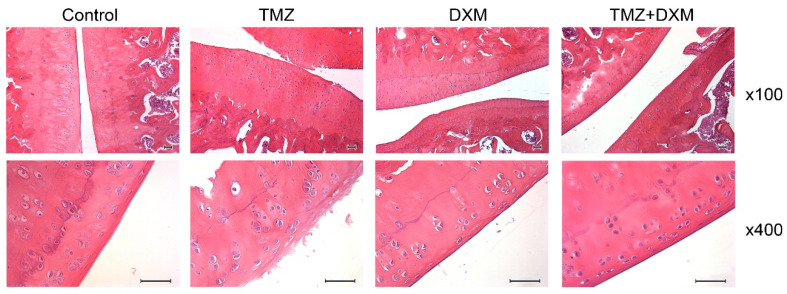
Histological analysis of the knee joint, fragments of the femoral and tibial epiphysis, and femur epiphyseal cartilage in the control, TMZ, DXM, and TMZ + DXM rat groups. Hematoxylin and eosin (H&E) staining. Magnification ×100, ×400. Scale bars 50 μm. TMZ—temozolomide, DXM—dexamethasone.

To investigate the potential molecular mechanism of the TMZ-induced decrease in locomotor activity of experimental animals, we performed a histological analysis of rats’ knee joints in all experimental groups, as the articular cartilage destruction can be directly associated with the locomotor changes observed. The knee joints formed by the proximal epiphysis of the tibia with a small area of the diaphysis, the distal epiphysis of the femur with a small area of the diaphysis, and the patella were analysed using hematoxylin and eosin (H&E) staining (Figure 3).

### 3.3. TMZ and DXM Affect on the Length and Ratio of Epiphyseal Cartilage Zones Knee Joint

The treatment of elderly experimental rats with TMZ led to degenerative changes in hyaline cartilage such as cartilage destruction, fragmentation, lesions, and delamination in the superficial epiphyseal cartilage of the femoral epiphysis, affecting the proliferative zone and the maturation cartilage zone. The organisation of chondrocytes into columns in the femoral epiphyseal plate was practically not preserved; the main matrix of the cartilage predominated (Figure 3, TMZ). Unlike TMZ, DXM did not affect the cartilage structure, and only some animals possessed light foci of cartilage loosening. Moreover, DXM was capable of preventing TMZ-induced cartilage degradation, the epiphyseal cartilage was basically normal, the surface layer consisted of 2–4 rows of young chondrocytes, and the upper rows of cells were more flattened (Figure 3, TMZ + DXM). These results correspond to those obtained in the open field test, demonstrating a toxic effect of TMZ on knee joint cartilage structure in experimental animals and the protective effect of DXM onto the joint knee structure.

### 3.4. TMZ and DXM Affect on the Length and Ratio of Epiphyseal Cartilage Zones of Knee Joint

To quantify the observed TMZ-induced changes in the rats’ knee joint, different parameters of the distal femoral epiphysis and proximal tibial epiphysis cartilage such as the number of chondrocytes (Table 1), percentage of bone marrow in the epiphysis and diaphysis of bones (Table 2), and analysis of the epiphyseal cartilage zones (Table 3) were studied. It was shown that neither TMZ nor DXM changed the concentration of chondrocytes in the epiphyseal cartilage and the epiphyseal plate of both the femur and tibia (Table 1) or the percentage of bone marrow in the epiphysis and diaphysis of the knee joints (Table 2).

However, long-term TMZ treatment selectively and significantly affected cartilage organisation in proximal tibial, but not distal femoral epiphysis (Table 3). The mean thickness of the proximal tibial epiphysis was decreased twofold (*p* < 0.001), whereas the percentage of proliferation zone was increased fourfold (*p* < 0.01) along with the corresponding decrease in the maturation zone of the cartilage. DXM administration did not affect the cartilage structure significantly, except for the decrease in mean thickness of epiphyseal cartilage (*p* < 0.05) of the proximal tibial epiphysis (Table 3).

Combined TMZ + DXM treatment effects were very similar to those after TMZ-only treatment and consisted of the increase of proliferation zone percentage (*p* < 0.01) and decrease in maturation zone percentage (*p* < 0.01), mean thickness of epiphyseal cartilage (*p* < 0.001), and mean thickness proliferative zone of epiphyseal cartilage (*p* < 0.01) of the proximal tibial epiphysis. In general, one can conclude that the identified changes in proximal tibial epiphysis are determined mainly by the effect of TMZ, which affects the knee joint cartilage structure during long-term chemotherapy.

### 3.5. TMZ and DXM Affect the Content and Localisation of GAGs in Rat Knee Joint Cartilage

**Figure 4:**
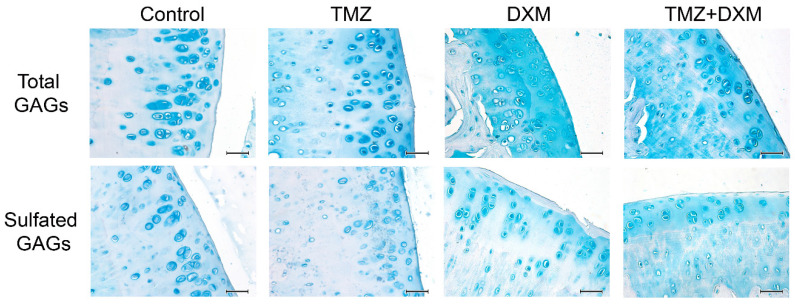
The content of GAGs in the knee joint cartilage of Wister rats treated with TMZ and/or DXM. Representative fragments of thefemoral and tibial epiphysis from the healthy (control), TMZ-, DXM-, and TMZ + DXM-treated experimental animals. Alcian blue staining for total GAGs (pH = 2.5) or sulfated GAG (sGAG, pH = 1.0). Magnification ×400. Scale bars 50 μm. TMZ—temozolomide, DXM—dexamethasone.

The demonstrated TMZ-induced decrease in knee joint cartilage thickness and reorganisation of its main functional zones (proliferation/maturation) suggest that some molecular changes occur in the basic characteristics of this tissue, which is mainly composed of sulfated/non-sulfated polysaccharide molecules called glycosaminoglycans (GAGs) or mucopolysaccharides. To test the assumption, we performed selective staining of sulfated and non-sulfated GAGs in the studied knee joint tissues using the Alcian blue staining kit (Figure 4).

Differential staining is provided owing to the preferential binding of Alcian blue with total GAGs at pH = 2.5 and with sulfated GAGs at pH = 1.0. In healthy rat cartilage tissue, GAGs are located mainly in chondrocytes and carry a marked amount of sulfate groups. TMZ treatment resulted in an increase of total GAGs content as well as their appearance in the tissue matrix, and the effect was even more pronounced after combined TMZ + DXM treatment. Staining for sulfated GAGs, on the contrary, demonstrated a decrease in their content in chondrocytes of all the treated cartilage tissues other than the intensive staining of the tissue matrix zone (especially for TMZ + DXM-treated tissues). These results suggest that long-term TMZ treatment affects GAG composition and/or localisation in knee joint cartilage of the elderly Wistar rats, and these changes might be of importance for locomotor activity of the experimental animals.

## 4. Discussion

In this work, the side effects of long-term administration of TMZ and/or DXM on the locomotor activity of elderly rats were studied. It has been shown that TMZ decreases horizontal motor activity; DXM treatment increases both horizontal and vertical motor activities, and long-time treatment with a combination of TMZ and DXM leads to an increase in the vertical motor activity of rats.

The effect of TMZ on various cognitive functions has been investigated previously in clinical studies (memory, attention, and overall life quality of patients) [15,16] and in animal models (parameters related to depression and anxiety) [17,18]. However, the effect of the drug on locomotor activity has not been studied enough, with research mainly focused on young animals [18,19]. According to previous studies, TMZ does not affect the locomotor activity of immature adult mice (7-week-old) in the open field test after administration of six3-day cycles of the drug for 6 weeks [18] or 8–12 weeks old mice after short-term TMZ treatment for 3 consecutive days [19]. Our data demonstrating the negative effect of long-term (12 weeks) TMZ administration on the physical activity of 9-month-old elderly rats extend the published results and suggest that the effect of TMZ on the animals’ locomotor activity may strongly depend on animal species and especially their age. We hypothesised that the negative effect of TMZ on the young mice’s locomotor activity and articular cartilage structure is being somehow compensated, whereas it becomes significant in the elderly animals.

The effect of DXM has been previously studied in both adult and newborn animals. DXM treatment of young rats for 9 days at a dose of 10 mg/kg significantly decreases horizontal and vertical locomotor activity of the animals in the open field test [30]. Long-term (6 weeks) low-dose (0.5 mg/kg) DXM administration results in a decrease in locomotor activity of 7-week-old rats [31]. According to our results, elderly Wistar rats (13 months old) demonstrate the opposite reaction to long-term DXM treatment (2.5 mg/kg) by increasing both horizontal and vertical locomotor activity. It seems that, in the elderly rats, DXM acts as “protector” for locomotor activity, increasing the motor function of the elderly animals.

Our data on the effect of long-term TMZ administration on the cartilage structure of elderly Wistar rats are difficult to compare with other data because similar studies have not been conducted before. A search for “temozolomide cartilage” in PubMed database returned one result, investigating the anti-proliferative effect of TMZ after unsuccessful surgery for herniated lumbar disc. In that work, TMZ was used as an agent preventing the development of excessive epidural fibrosis—the excessive scar formation near the root of a nerve. Adult rats underwent L3 and L4 laminectomy, and then TMZ was administered at a dose of 18 mg/kg for 5 days after the surgery. TMZ administration did not affect bone and cartilage regeneration, arachnoidal fibrosis, and inflammatory and fibroblast cell densities; however, it led to a decrease in epidural fibrosis [32]. There are no data explaining in detail the mechanism of such a negative effect of TMZ on cartilage structure. In our work, it was shown that TMZ leads to degenerative changes in the epiphyseal cartilage of rat knee joint, which may be one of the possible mechanisms for reducing the locomotor activity of elderly animals.

As for DXM, previous studies were also mainly focused on its effects on the morphological structure and ultrastructure of the cartilage of healthy young animals. According to our results, long-term DXM administration leads to a decrease in mean thickness of epiphyseal cartilage (*p* < 0.05) of the proximal tibial epiphysis, which is consistent with the results obtained at another object—pony foals (6 months old), according to which 3–11 months’ DXM administration did not affect the cartilage structure significantly, altering only the mean thickness of epiphyseal cartilage of the proximal tibial epiphysis [33]. However, the observed changes in the organisation of chondrocytes in the epiphyseal plate of the pony foals (increased spatial separation between chondrocyte columns, narrowed zones of disorganised columnar, and hypertrophic cartilage) [33] are not observed in the cartilage of elderly rats, supporting evident species- and age-dependency of DXM effects on cartilage structure. According to data from other studies, degenerative changes in the cartilage of the knee joints of adult rats are accompanied by ultrastructural changes; that is, a decrease in the amount of rough endoplasmic reticulum and Golgi apparatus, an increase in glycogen content after 5 weeks of DXM administration [34], an increase in cell mortality rate, a decrease in the length of endoplasmic reticulum, a decrease in the number of Golgi apparatus, and a decrease in mitochondrial pool and size after 3 weeks of DXM administration [35]. Taken together, the results on the effects of DXM on the articular cartilage in healthy animals are controversial; DXM has been shown to affect cartilage and chondrocytes depending on the treatment duration [36].

In the literature, there are no articles on the study of changes in the GAGs content in cartilage after TMZ administration. We have shown for the first time that the long-term administration of TMZ to elderly Wistar rats leads to an increase in the total content of GAGs and the redistribution of sulfated GAGs between chondrocytes and the matrix of the epiphyseal cartilage of the knee joints. It is interesting that similar changes in GAGs content were observed in the rat brain tissue, where TMZ increases the CS content (one of the main classes of GAGs) in the cerebral cortex of rats after 5 days of drug administration [37].

There is no consensus in the literature on the effect of DXM on the GAG content in cartilage. One of the mechanisms of the corticosteroids’ effects may be associated with changes in GAGs. The GAG content seems to be increased after DXM injection by reducing the activity of extracellular degradation enzymes [36]. However, according to another work, 9-week administration of other corticosteroid drug cortisone to adult rabbits led to a gradual decrease in the component of GAG-the hexosamine concentration content in articular cartilage [38] and a 55% decrease in GAG content in epiphyseal and articular cartilages of pony foals knee joint after 3 months of treatment [33]. In addition, the administration of DXM together with exogenous glycosaminoglycan-peptide-complex was capable of normalising most of the DXM-induced ultrastructural changes and protecting cartilage from the inhibitory effects of corticoids [35].

The study of the effect of the DXM/TMZ combination on GAG content in cartilage has not been previously investigated. However, there are data on the effect of a combination of these drugs on the healthy rat brain tissue in which the amount of CS was increased in the rat hippocampus and cortex after one week of administration of TMZ and DXM [37]. According to our data, TMZ demonstrates a negative effect on the morphology of the elderly rat knee joints, whereas DXM significantly affects the total GAGs content in the cartilage tissue.

## 5. Conclusions

Taken together, the obtained results demonstrate that long-term administration of the chemotherapeutic drug temozolomide leads to deferred changes in locomotor activity of elderly experimental Wistar rats, disorganisation of proximal tibial epiphysis, and changes in GAG composition/localisation in knee joint cartilage.

## Figures and Tables

**Table 1:** The number of chondrocytes per unit area of the Wistar rat knee joint cartilage, units/(µM^2^ × 10^4^). All data are expressed as mean ± SD (ORIGIN 8.5 software). TMZ—temozolomide, DXM—dexamethasone.

Group	Distal Femoral Epiphysis		Proximal Tibial Epiphysis	
	Epiphyseal Cartilage, Maturation Zone	Epiphyseal Plate	Epiphyseal Cartilage, Maturation Zone	Epiphyseal Plate
Control	20.35 ± 4.43	15.91 ± 1.11	22.17 ± 0.24	20.93 ± 2.88
TMZ	24.12 ± 2.37	1332 ± 1.56	22.98 ± 1.07	18.54 ± 1.21
DXM	20.05 ± 3.02	13.14 ± 0.72	23.09 ± 3.19	17.37 ± 1.45
TMZ + DXM	23.61 ± 1.3	17.82 ± 2.66	24.11 ± 2.92	25.18 ± 1.17

**Table 2:** The percentage of bone marrow in the epiphysis and diaphysis of the knee joint Wistar rat bones.All data are expressed as mean ± SD (ORIGIN 8.5 software). TMZ—temozolomide, DXM—dexamethasone.

Group	Femur Bone		Tibia Bone	
	Primary Ossification Center (Diaphysis)	Secondary Ossification Center (Epiphysis)	Primary Ossification Center (Diaphysis)	Secondary Ossification Center (Epiphysis)
Control	78.52 ± 4.13	64.05 ± 8.75	86.96 ± 2.71	68.64 ± 5.54
TMZ	76.21 ± 4.49	58.64 ± 5.97	75.96 ± 5.13	62.00 ± 5.44
DXM	86.19 ± 1.97	62.12 ± 1.41	81.65 ± 1.27	71.71 ± 1.90
TMZ + DXM	83.11 ± 6.05	65.93 ± 3.68	80.35 ± 3.32	60.23 ± 5.86

**Table 3:** Quantitative analysis of the epiphyseal cartilage zones of the Wistar rats knee joint. All data were expressed as mean ± SD (ORIGIN 8.5 software). * *p* < 0.05, ** *p* < 0.01, *** *p* < 0.001 compared with control. TMZ—temozolomide, DXM—dexamethasone.

Group	Distal Femoral Epiphysis				Proximal Tibial Epiphysis			
	Length of Proliferation Zone/Maturation Zone, %	Length of Maturation Zone/Epiphyseal Cartilage, %	Mean Thickness of Epiphyseal Cartilage, µM	Mean Thickness of Epiphyseal Cartilage Proliferative Zone, µM	Length of Proliferation Zone/Maturation Zone, %	Length of Maturation Zone/Epiphyseal Cartilage, %	Mean Thickness of Epiphyseal Cartilage, µM	Mean Thickness of Epiphyseal Cartilage Proliferative Zone, µM
Control	18.03 ± 2.86	36.44 ± 1.68	195.83 ± 37.65	12.23 ± 1.23	6.86 ± 0.13	65.68 ± 0.07	349.08 ± 14.90	15.71 ± 0.35
TMZ	18.58 ± 2.33	37.25 ± 1.47	198.39 ± 33.81	13.63 ± 2.92	25.40 ± 4.91 **	43.92 ± 4.56 **	161.63 ± 40.26 ***	17.07 ± 2.04
DXM	21.34 ± 4.99	37.77 ± 5.84	210.73 ± 11.48	15.82 ± 2.24	10.29 ± 1.18	56.01 ± 3.48	266.37 ± 29.30 *	14.70 ± 1.65
TMZ + DXM	17.24 ± 4.88	38.69 ± 4.30	228.39 ± 31.91	13.54 ± 0.72	15.51 ± 1.50 **	36.73 ± 5.77 **	208.53 ± 28.67 ***	11.32 ± 0.87 **
